# Differential Gene Expression Changes in Human Primary Dental Pulp Cells Treated with Biodentine and TheraCal LC Compared to MTA

**DOI:** 10.3390/biomedicines8110445

**Published:** 2020-10-22

**Authors:** Ok Hyung Nam, Ho Sun Lee, Jae-Hwan Kim, Yong Kwon Chae, Seoung-Jin Hong, Sang Wook Kang, Hyo-Seol Lee, Sung Chul Choi, Young Kim

**Affiliations:** 1 Department of Pediatric Dentistry, School of Dentistry, Kyung Hee University, Seoul 02447, Korea; pedokhyung@gmail.com (O.H.N.); imhosun@khu.ac.kr (H.S.L.); pedochae@gmail.com (Y.K.C.); snowlee@khu.ac.kr (H.-S.L.); pedochoi@khu.ac.kr (S.C.C.); 2 Department of Pediatric Dentistry, School of Dentistry, Jeonbuk National University, Jeonju 54896, Korea; jhbcss@hanmail.net; 3 Department of Prosthodontics, School of Dentistry, Kyung Hee University, Seoul 02447, Korea; ssabock@hanmail.net; 4 Department of Oral and Maxillofacial Pathology, School of Dentistry, Kyung Hee University, Seoul 02447, Korea; pathemis@khu.ac.kr; 5 Department of Oral Pathology, School of Dentistry, Chonnam National University, Gwangju 61186, Korea

**Keywords:** gene expression, mineral trioxide aggregate, Biodentine, TheraCal LC, direct pulp capping, primary tooth

## Abstract

This study aimed to analyze the effects of pulp capping materials on gene expression changes in primary tooth-derived dental pulp cells using next-generation sequencing. Dental pulp cells were extracted and treated with mineral trioxide aggregate (MTA), Biodentine (BD), or TheraCal LC (TC). Cell viability assays were performed. Total RNA was extracted and analyzed through mRNA sequencing. Bioinformatic analysis of differential gene expression in dental pulp cells exposed to BD or TC versus MTA was performed. MTA, BD, and TC exposure had no significant effect on pulp cell viability (*p* > 0.05). Gene sets associated with inflammatory response (*p* = 2.94 × 10^−5^) and tumor necrosis factor alpha (TNF-α) signaling via the nuclear factor kappa-light-chain-enhancer of activated B cells (NF-κB) pathway (*p* = 2.94 × 10^−5^) were enriched in all materials. In BD-treated cells, Wnt/β-catenin signaling (*p* = 3.15 × 10^−4^) gene sets were enriched, whereas enrichment of interferon gamma (IFN-γ) response (*p* = 3 × 10^−3^) was observed in TC-treated cells. In gene plot analysis, marked increases in receptor activator of nuclear factor kappa-Β ligand (*RANKL*) expression were seen in TC-treated cells over time. Despite the similar cell viabilities exhibited among MTA-, BD-, and TC-treated cells, patterns of gene networks differed, suggesting that diverse functional gene differences may be associated with treatment using these materials.

## 1. Introduction

Direct pulp capping (DPC) is a biological treatment used to ensure pulp vitality by sealing the pulp exposure site with a biomaterial [1]. Previously, DPC was not recommended in primary teeth, as its success rate was considerably low [2,3]. A previous study identified the presence of a cluster of CD34^+^ cells in the primary tooth pulp by microscopic analysis, and demonstrated these are undifferentiated mesenchymal-like cells [4]. Another study suggested that the low success rate of DPC in primary teeth is due to the large number of undifferentiated mesenchymal stem cells in the pulp of primary teeth [2]. These stem cells can differentiate not only into odontoblasts, but also into odontoclasts, which may hamper pulp healing. Therefore, an ideal goal of DPC should be to promote differentiation of undifferentiated stem cells into odontoblastic cells instead, which promote pulp regeneration via hard tissue formation [5]. To facilitate hard tissue formation, great attention has been focused on the interaction between pulp cells and pulp capping materials.

Recently, various pulp capping materials have been proposed for use in DPC of primary teeth [1,6,7]: in particular, mineral trioxide aggregate (MTA) has served as the gold standard because of its excellent antibacterial performance and biocompatibility [8]. However, long setting times and low resistance to discoloration remain major drawbacks of MTA [9,10], which were addressed with the more recently developed Biodentine (BD) and TheraCal LC (TC). BD is a calcium silicate cement that has a shorter setting time and less discoloration potential compared to MTA [9,11]. TC is composed of resin monomers and type III Portland cement. Thus, the setting time can be accelerated by light-curing. TC releases calcium ions after light-curing and has good bioactivity [12]. These advantages have resulted in more widespread use of BD and TC as pulp capping materials for DPC in primary teeth [1,6,7,13].

Previous in vitro studies confirmed that MTA stimulates odontogenic differentiation, proliferation, and mineralization of human dental pulp stem cells (hDPSCs) [14,15]. A previous microarray study reported that MTA positively affects genetic changes in hDPSCs [16]. The study concluded that MTA greatly influences mineralization and induces slight inflammation. In an in vitro DPC model, BD showed the potential to preserve hDPSC proliferation, migration, and adhesion [17]. Moreover, previous studies comparing the effects of MTA and BD reported that BD accelerated hDPSC proliferation in comparison to MTA [18,19]. TC has been shown to result in superior cell viability of hDPSCs compared to calcium hydroxide [20]. Conversely, TC has also been reported to have low cytocompatibility compared to MTA and BD [21]. A similar phenomenon was confirmed by another study, which compared the cytotoxicity of resin-based sealers on hDPSCs and demonstrated that the cell viability was significantly lower for up to 72 h when resin-based sealers were used than that in the control group [22].

To date, several in vitro studies using real-time polymerase chain reaction (RT-PCR) have identified specific hDPSC gene expression regarding the biological effects of pulp capping materials. Sun et al. [23] evaluated the expression of alkaline phosphatase (*ALP*), collagen type I (*COL1*) and osteocalcin (*OCN*) after BD and iRoot Fast Set exposures. They demonstrated that there were no differences in the expression of these genes between the two materials. Yu et al. [24] evaluated an experimental DPC material composed of antibacterial resin monomers and Portland cement, and they concluded that the material promoted odontogenic differentiation through overexpression of odontogenic differentiation-related markers. Furthermore, in vitro studies using RNA sequencing are increasing. Lu et al. [25] assessed the gene expression profiles of hDPSCs and supernumerary teeth-derived stem cells and concluded that supernumerary teeth-derived stem cells had greater potential for cell differentiation.

Although the effects of pulp capping materials on hDPSCs are widely known, little is known about the effect of pulp capping materials on pulp cells in primary teeth. Considering the increasing clinical use of these materials for DPC of primary teeth, the response of human primary tooth pulp cells treated with recently developed pulp capping materials should be examined. Therefore, the aim of this study was to investigate the differential gene expression in BD- and TC-treated human primary tooth dental pulp cells and to compare them with MTA-treated cells using next-generation sequencing analysis.

## 2. Experimental Section

### 2.1. Preparation of Dental Pulp Cells from Human Primary Teeth

This study protocol was reviewed and approved (03 September 2020) by the Ethics Committee of Kyung Hee University Dental Hospital, Kyung Hee University, Seoul, Korea (KH-DT20021). Dental pulp samples were obtained from three primary teeth that were physiologically exfoliated from healthy children. Briefly, pulp tissues were carefully extracted from the teeth and immediately placed in phosphate-buffered saline (PBS) solution (WELGENE, Gyeongsan-si, Geyongsangbuk-do, Korea). Extracted tissues were cut into small pieces and preserved in a growth medium composed of alpha minimum essential medium (α-MEM, Gibco Invitrogen, Grand Island, NY, USA), 10% fetal bovine serum (FBS), 100 U/mL penicillin, and 100 mg/mL streptomycin. Cells were seeded into 6-well plates and incubated in a 37 °C, in 5% carbon dioxide (CO_2_) and humidified atmosphere. As soon as cells became confluent, they were passaged four times using trypsin and only then used for experiments.

### 2.2. Material Preparation

Material preparation was performed as previously described [26]. MTA (ProRoot MTA; Dentsply, Tulsa Dental, Tusal, OK, USA), BD (Septodont, Saint-Maur-des-Fossés, France), and TC (Bisco, Schaumburg, IL, USA) were prepared under aseptic conditions. MTA and BD were mixed in accordance with the manufacturers’ instructions, loaded into a polyethylene tube (5 mm diameter, 3 mm height) for molding, and allowed to set for 24 h at 37 °C in a humidified atmosphere. TC was loaded into a polyethylene tube, light-cured for 120 s, and then allowed to set for 24 h at 37 °C in a humidified atmosphere. Ultraviolet radiation was used for sterilization. Growth medium (50 mL) was added to the molded materials and allowed to infuse. A 1:20 dilution of growth medium infused with the materials was prepared before the experiments and filtered using a 0.2 μm syringe filter.

### 2.3. Cell Viability Assay

Cell viability was evaluated using a water-soluble tetrazolium salt assay. Donor cells were pooled, seeded in 96-well plates (2 × 10^4^ cells/well) in α-MEM supplemented with 10% FBS, and incubated for 24 h. Cells were then treated with a 1:20 dilution of growth medium infused with the pulp capping materials and left to incubate for either 24 or 72 h; 10 µL tetrazolium salt reagent (EZ-CYTOX; Daeil Lab, Seoul, Korea) was added to each well before incubating the cells at 37 °C for further 4 h. The optical density (OD) was read at 450 nm using a Multiskan GO microplate spectrophotometer (Thermo Scientific, Waltham, IL, USA). Cell viability was calculated using the formula: OD (experimental material) / OD (control; no treatment) and presented as a percentage.

### 2.4. Next-Generation Sequencing

Total RNA was extracted using TRIzol Reagent^TM^ (Invitrogen, Carlsbad, CA, USA) according to the manufacturer’s instructions. RNA quality and quantity were measured using an Agilent 2100 bioanalyzer with an RNA 6000 Nano LapChip kit (Agilent Technologies, Amstelveen, The Netherlands) and an ND-2000 Spectrophotometer (Thermo, Wilmington, DE, USA). All RNA libraries were constructed using the QuantSeq 3 mRNA-Seq Library Prep Kit (Lexogen, Vienna, Austria). Polymerase chain reaction was performed for RNA purification using PCR Mix and Enzyme Mix 3 kit (Lexogen, Vienna, Austria). The reactions were optimized by examining the thermocycling temperature (72–98 °C) and time (10–30 s), cooling temperature (10 °C), and the number of PCR cycles (16 cycles). Next, high-accuracy sequencing was assessed by high-throughput, single-end 75 bp sequencing using a NextSeq 500 (Illumina, San Diego, CA, USA).

### 2.5. Identification of Differentially Expressed Genes (DEGs)

Bowtie2-build [27] was used to align the QuantSeq 3 mRNA-Seq reads to the human reference genome. Bowtie2 indexes were generated from either genome assembly sequences or representative transcript sequences that aligned to the genome and transcriptome. Aligned reads were used for assembly and quantification of transcripts and identification of differential gene expression. DEGs were determined based on the read counts using coverage in BEDtools [28]. Quantile normalization of the read count data was performed using EdgeR within R software (R Development Core Team, Vienna, Austria) [29].

Gene expression was determined using the ExDEGA 1.2.1.0 program (EBIOGEN, Seoul, Korea). Only genes with greater than 2-fold change in expression compared to MTA (set as control) and with a *p*-value < 0.05 were considered to be DEGs, as previously described [26]. The Database for Annotation, Visualization, and Integrated Discovery (DAVID v6.8) (http://david.abcc.ncifcrf.gov) was used to search for gene classification.

### 2.6. Bioinformatics Analysis

Hallmark gene sets were generated using gene set enrichment analysis (GSEA) 4.0.0. program [30]. The false discovery rate (FDR) was set at *q* < 0.05 [31]. Gene plot analyses were performed using ExDEGA. To analyze the molecular interactions of cell proliferation, differentiation, and migration, the Search Tool for the Retrieval of Interacting Genes (STRING) was used to generate protein-protein interaction (PPI) networks with a 2-fold change cutoff. The minimum required interaction score was set at a high confidence level (>0.700). The number of clusters was set at 3 for k-means clustering.

### 2.7. Statistical Analysis

SPSS 20.0 (SPSS, Chicago, IL, USA) software was used for statistical analysis. The Kruskal-Wallis test and Mann-Whitney test (as post hoc analysis) were used for cell viability and the results were only considered statistically significant if *p* < 0.05.

## 3. Results

### 3.1. Cell Viability

**Figure 1:**
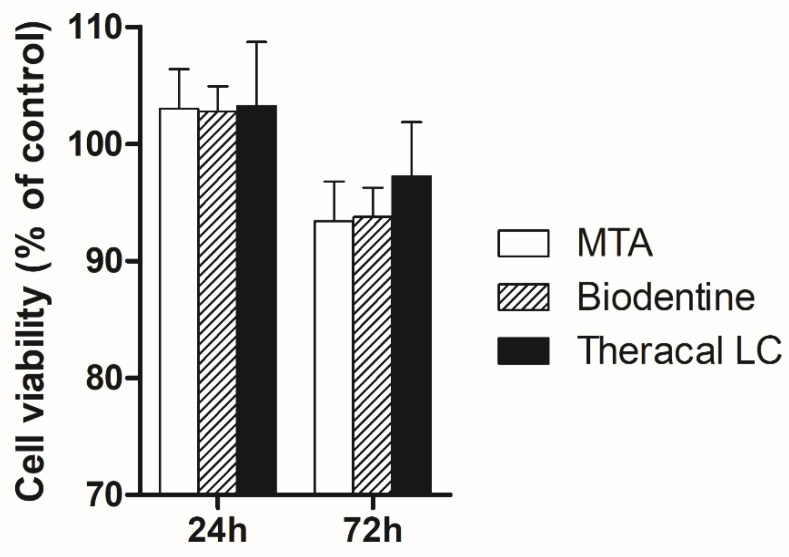
Cell viability test. The cell viability under different conditions was evaluated by water-soluble tetrazolium salt assay. There were no significant differences among different conditions both 24 and 72 h (*p* > 0.05). Control, no treatment.

To evaluate the effect of pulp capping materials on cell viability, cells in 96-well plates were treated with MTA, BD, and TC conditioned growth medium, and the OD of these wells was compared. As shown in Figure 1, compared with that in MTA, cell viabilities in the BD and TC groups were not significantly decreased (*p* > 0.05). With time, the cell viabilities in the presence of MTA, BD, and TC decreased without statistical significance (*p* > 0.05).

### 3.2. DEG Identification

**Figure 2:**
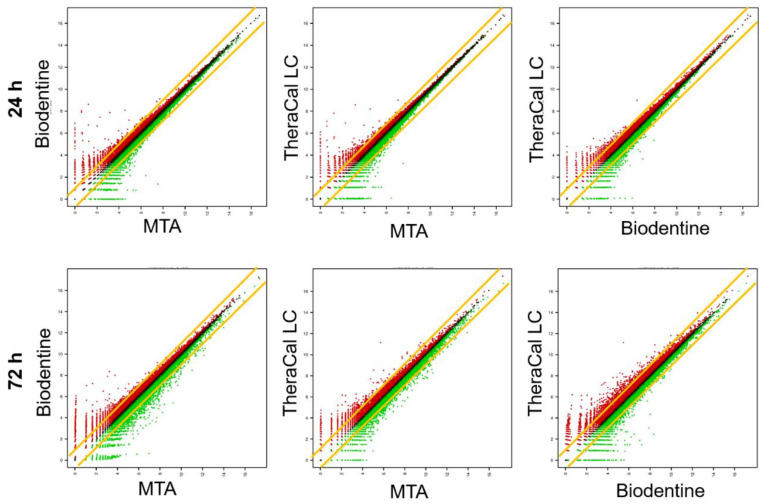
Scatter plots of gene expression. In each plot, the central line passing through the origin indicates no difference in expression between the cells stored for different time periods. Any plot outside the yellow line indicates that there is more than a 2-fold difference. Red plots represent up-regulated genes, while green plots present down-regulated genes.

**Figure 3:**
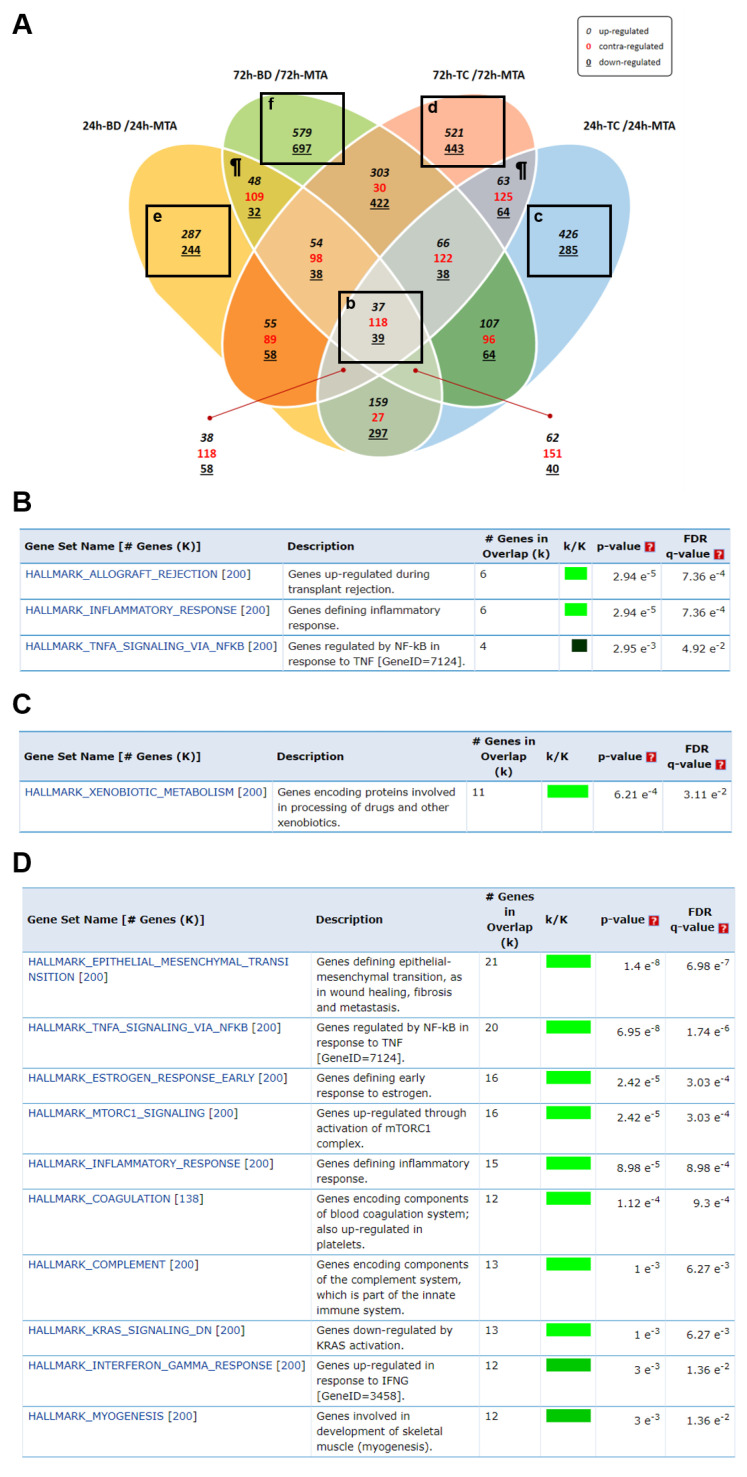

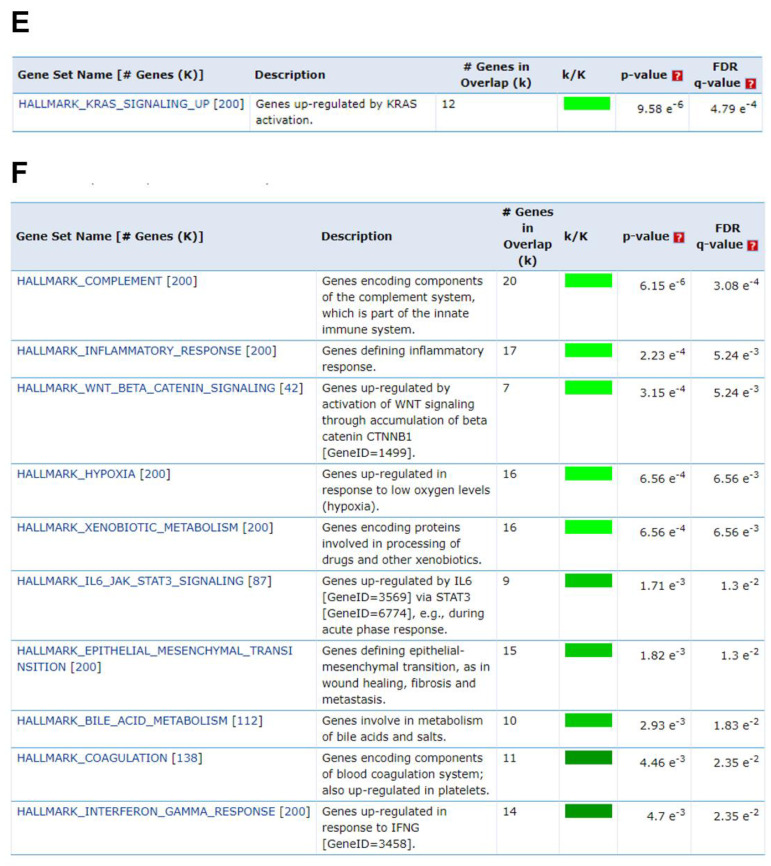
Gene set enrichment analysis. (**A**) Venn diagram analysis. (**B**–**F**) Hallmark gene set enrichment analysis. Only gene sets with *q*-value < 0.05 were included. (**B**–**F**) show detailed data of the boxes with the respective lowercase letters in (**A**). No significant gene sets were screened in the hallmark gene set analysis; FDR, false discovery rate; k/K, Color bar shading from light green to black, where lighter colors indicate more significant FDR *q*-value (<0.05) and black indicates less significant FDR *q*-values (≥0.05).

Scatter plot analysis showed that the DEG profiles of MTA-, BD-, and TC-treated cells were predominantly overlapping (Figure 2). Although not statistically significant, the DEG profiles of BD- and TC-treated cells appeared to differ the most in cells treated for 72 h. Figure 3A shows a Venn diagram of DEGs in BD- and TC-treated cells compared to MTA-treated cells. There was an overlap of 194 DEGs between BD- and TC-treated cells, with 37 up-regulated genes, 39 down-regulated genes, and 118 contra-regulated genes.

### 3.3. Gene Set Enrichment Analysis (GSEA)

Figure 3B shows hallmark gene set enrichment in both BD-treated and TC-treated cells at all treatment durations. The enriched gene sets were associated with allograft rejection, inflammatory responses, and TNF-α signaling via the NF-κB pathway. Interestingly, these hallmark gene sets were enriched only in contra-regulated DEGs. Table 1 shows the list of overlapping DEGs in these hallmark gene sets. There were different levels of overlapping DEGs from BD- and TC-treated cells compared to MTA-treated cells (except for *KLRD1*) over time. For example *CCL5* expression in MTA-treated cells increased over time, whereas *CCL5* expression in BD- and TC-treated cells decreased over time.

Among the hallmark gene sets only enriched in TC-treated cells, xenobiotic metabolism was identified in cells treated for 24 h (Figure 3C). TNF-α signaling via the NF-κB pathway, inflammatory responses, and INF-γ responses were enriched in cells exposed to TC for 72 h (Figure 3D). Hallmark gene sets only enriched in BD-treated cells at 24 h included KRAS signaling (Figure 3E). In BD-treated cells, inflammatory responses, Wnt/β-catenin signaling, interleukin 6/Janus kinases/signal transducer and activator of transcription proteins 3 (IL-6/JAK/STAT3) signaling, and INF-γ responses were enriched after 72 h (Figure 3F).

### 3.4. Gene Plot Analysis

**Figure 4:**
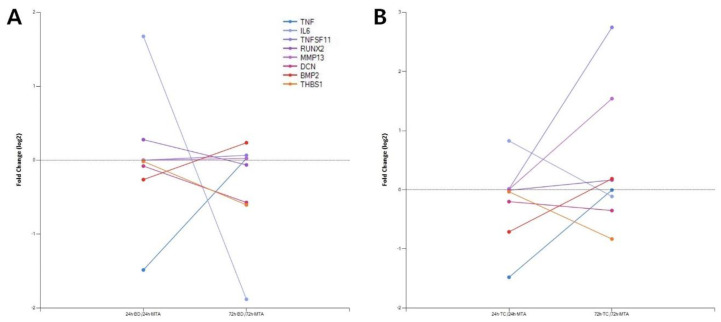
Gene plot analysis. Expression of specific genes in Biodentine- and TheraCal LC- were compared to that in MTA-treated cells. (**A**) Biodentine. Please note that *BMP2* expression was down-regulated at 24 h and up-regulated at 72 h. (**B**) TheraCal LC. Please note that marked increase of *RANKL* expression in 72 h compared to 24 h. TNF, *TNF-α*; IL6, *IL-6*; TNFSF11, *RANKL*.

Several genes related to inflammatory response (*TNF-α*, *IL-6*), odontogenesis (*RUNX2*, *MMP-13*), osteoclastogenesis (*RANKL*), and mineralization (*BMP2*, *THBS1*) were included in the gene plot analysis (Figure 4). *TNF-α* expression increased in both BD- and TC-treated cells. *IL-6* expression was down-regulated in BD-treated cells compared to that in MTA from 24 h to 72 h. *RANKL* expression decreased in BD-treated cells, whereas *RANKL* expression in TC-treated cells increased greatly from 24 h to 72 h. In addition, genes related to mineralization showed weak expression in both BD- and TC-treated cells at both time points, except for Decorin (*DCN*) expression in BD-treated cells.

### 3.5. PPI Network Analysis

**Figure 5:**
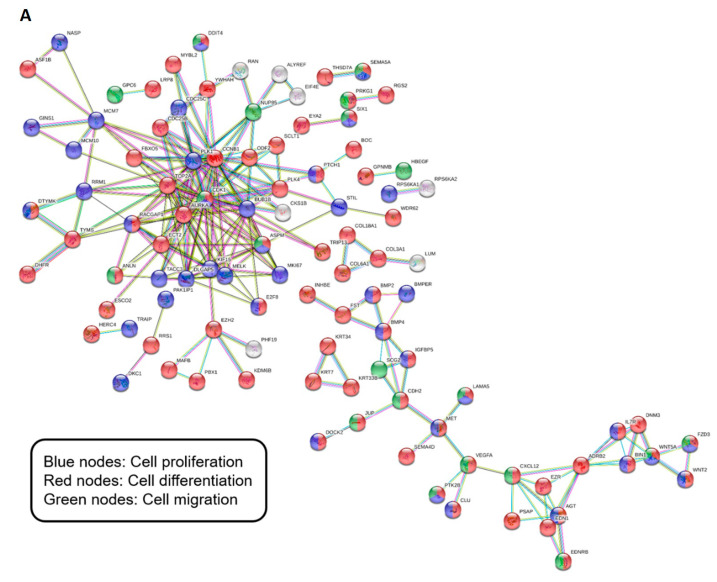

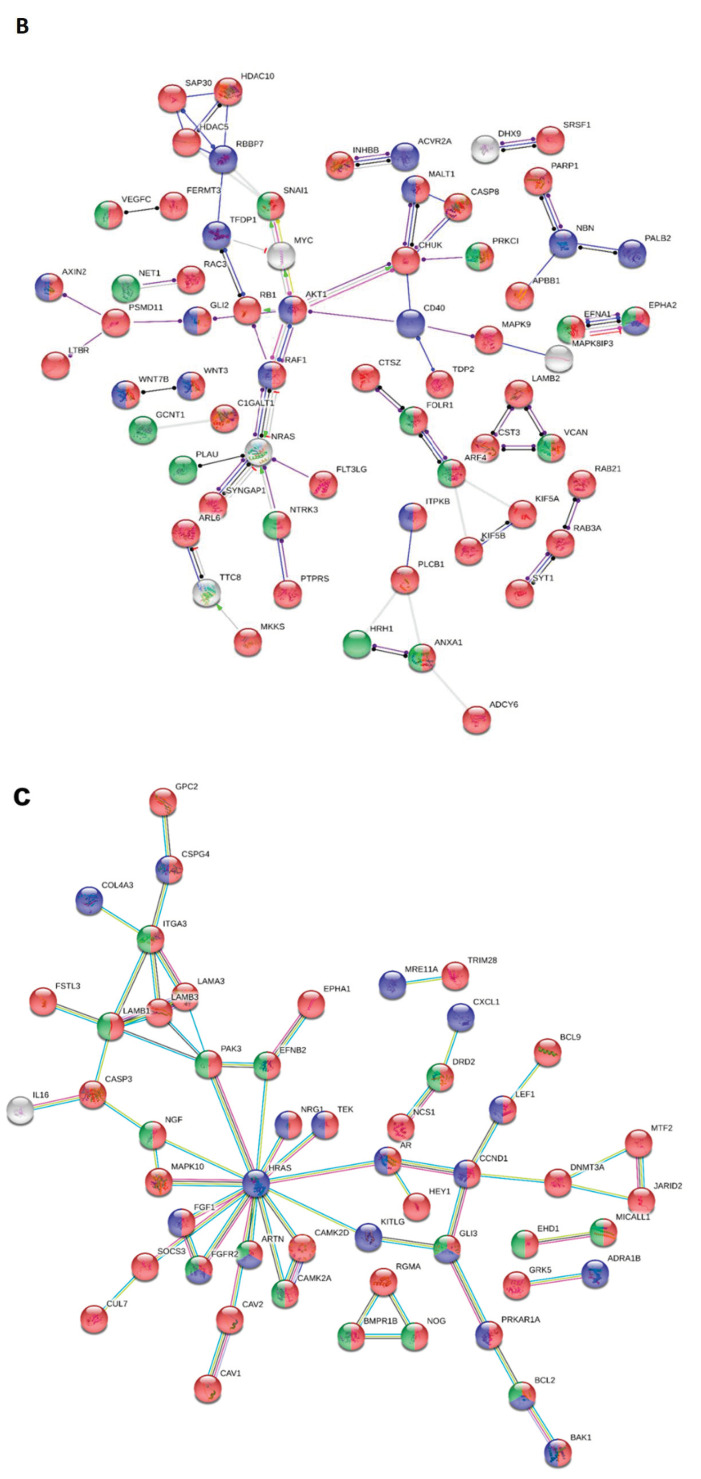
Gene network construction. (**A**) Gene networks significantly expressed in the cells exposed to all materials. (**B**) Gene networks significantly expressed in the cells exposed to Biodentine. (**C**) Gene networks significantly expressed in the cells exposed in TheraCal LC. Disconnected proteins are hidden. Blue nodes, genes associated with cell proliferation; red nodes, genes associated with cell differentiation; green nodes, genes associated with cell migration.

For the DEGs expressed in all capping materials, the PPI network contained 198 nodes and 255 edges (Figure 5A). The PPI enrichment *p*-value was 1.0 × 10^−16^. The PPI network of the DEGs in BD-treatment only had 146 nodes and 63 edges, with *p*-value 0.0208 (Figure 5B). Figure 5C shows the PPI network of the DEGs in TC treatment only, with 147 nodes, 59 edges, and *p*-value 2.24 × 10^−7^.

## 4. Discussion

DPC of primary teeth is increasingly being applied in dental settings. Critical for the success of DPC is the selection of proper pulp capping materials to promote dentin bridge formation, as well as overlay restorations [32], which should have superior properties against bacterial penetrations [33]. Moreover, adequate bonding between the pulp capping materials and the restorations can provide adhesive joints and thus stress relief [34]. Recent clinical studies investigating pulp capping materials such as MTA and TC demonstrated high reliability using DPC in primary teeth [1,6,7,13].

Cell viability assays are usually performed to assess the biocompatibility of different pulp capping materials. In this study, cell viability after exposure to different pulp capping materials was well maintained for up to 72 h and was not affected by the type of capping material. These results are consistent with those of previous studies, suggesting that the viability of stem cells from human exfoliated deciduous teeth (SHED) treated with MTA and BD are similar after 72 h [35].

Scatter plots showed that the DEG profiles were similar in cells treated with the different materials. However, considerable differences in gene expression were observed at 72 h between TC-, MTA-, and BD-treated cells. These findings suggest that further biological differences may emerge over a longer period of time in cells exposed to TC compared to those exposed to MTA or BD.

To thoroughly investigate the functional differences in gene expression between BD- and TC-treated cells compared to MTA-treated cells over time, hallmark GSEA was performed. The hallmark gene sets enriched in cells exposed to each of the materials were inflammatory response pathways and TNF-α signaling via the NF-κB signaling pathways. The pulpal defense and repair mechanisms against dentin-invading bacteria involve secretion of pro-inflammatory and immunomodulatory mediators by odontoblasts into the pulp area, resulting in the activation of various immune cells [36]. NF-κB is an important transcription factor in this mechanism [37].

Expression of *CCL5*, *IL-18*, *ICOSLG*, and *CXCL6* overlapped in the two enriched pathways (Table 1). Interestingly, there were differences in gene expression levels over time between MTA-treated cells compared to BD- and TC-treated cells. This finding suggests that the biological functions of these hallmark pathways may be different in MTA-treated cells compared to BD- and TC-treated cells. *CCL5* is a pro-inflammatory cytokine induced by NF-κB activation. In this study, MTA-treatment resulted in decreased *CCL5* expression over time. This result is consistent with that of a previous study, suggesting that MTA has an anti-inflammatory effect via *CCL5* down-regulation [38]. However, BD and TC treatment resulted in increased *CCL5* expression over time. *IL-18* is also a pro-inflammatory cytokine, and its secretion is strongly induced in pulp inflammation caused by dental caries [39]. *IL-18* expression in MTA-treated cells increased over time, whereas BD and TC treatment resulted in decreased *IL-18* expression. *CXCL6* is a pro-inflammatory cytokine associated with chemotactic and pro-angiogenic activity: a human study on apical periodontitis reported that its expression originates from inflamed gingival tissue [40], while another study reported its overexpression (fold change >100) in the odontoblastic layer of carious teeth [41].

Wnt/β-catenin signaling has shown potential to enhance odontoblastic differentiation in hDPSCs [42]. Interestingly, gene sets associated with Wnt/β-catenin signaling were enriched in BD-treated cells at 72 h. This finding is consistent with a previous study on DPC in rat molars [43], which observed more favorable treatment outcomes in molars treated with BD than with MTA. The study reported that β-catenin expression was only observed in BD-treated rat molars and suggested that Wnt/β-catenin signaling may be related to BD-induced reparative dentin formation.

IFN-γ mediates innate and adaptive immune responses [44] and is necessary for osteogenic differentiation of mesenchymal stem cells [45]. A recent study reported that high concentrations of IFN-γ positively affected dentinogenic functions in irreversible pulpitis-derived hDPSCs [46]. There is also evidence that IFN-γ is responsible for reparative dentin formation. [47]. Our results showed up-regulation of IFN-γ response gene sets in both BD- and TC-treated cells at 72 h.

Gene plot analysis was used to compare time-dependent gene expression changes in BD- and TC-treated cells compared to MTA-treated cells. Among the genes related to inflammatory responses, the expression pattern of *TNF-α* and *IL-6* differed. The intensity of *TNF-α* expression after 72 h in BD- and TC-treated cells was similar to that in MTA-treated cells. However, *IL-6* expression in BD-treated cells was down-regulated compared to those treated with MTA alone. Similar findings were confirmed by a study evaluating the effects of biomaterials on hDPSCs from apical papilla [48]. Interestingly, marked increases in *RANKL* expression were seen in TC-treated cells over time. *RANKL* is associated with osteoclastogenesis [49]. This finding confirms previous findings that TC-induced DEGs may be involved in the biological process associated with osteoclastogenesis [26]. Resin monomers have limited potential in promoting human dental pulp cells to differentiate into odontoclasts [50,51]. A previous in vitro study demonstrated that the odontoclastic differentiation ratios were related to resin monomers in a dose-dependent manner [50]. Because TC is cured by light, some resin components in the TC may be insufficiently polymerized and consequently released, thus exerting harmful effects on dental pulp cells [52]. Other adverse effects may include increased pulpal temperature during light-curing: temperature changes during light-curing can affect pulpal blood microcirculation [53]. Moreover, there is evidence that light-curing of pulp capping materials can cause deformation of pulpal dentin [54]. Thus, it appears that both resin monomers and light-curing could be responsible for the different biological effects of TC against those of MTA and BD. *BMP2* expression was also found to be highly up-regulated in BD-treated cells at 72 h, consistent with a previous study using human osteoblast cells [55].

To understand the comprehensive gene interactions between DEGs, we performed a PPI network analysis. As discussed, various genes identified in the same hallmark gene sets can differentially regulate specific signaling pathways. The PPI results showed complex gene interactions involving various cellular mechanisms. These findings support the concept that evaluation of the networks of various genes is essential, rather than evaluation of the function of individual genes [56,57].

Several studies have compared the biological effects of pulp capping materials on pulp cells [24,58]. However, these studies were limited to evaluating the expression of specific genes based on biological effects only. Instead, this study offers significant evidence to prove that analysis of gene-gene interactions, especially through GSEA and PPI network analysis, can provide a deeper and more comprehensive understanding of the effects of pulp capping materials on pulp cells.

## 5. Conclusions

In conclusion, this study evaluated the DEG profiles of primary tooth dental pulp cells treated with different pulp capping materials using next-generation sequencing. DEGs in MTA-, BD-, and TC-treated cells were largely overlapping. However, the regulation of overlapping DEGs differed among the pulp capping materials examined. This finding can provide a new insight into the biological effects of pulp capping materials.

## Figures and Tables

**Table 1:** Overlapping differentially expressed genes (DEGs) in hallmark gene sets enriched in all pulp capping materials.

Gene	Description	Normalized Fold Change (log_2_)								
		MTA			BD			TC		
		24 h	72 h	G	24 h	72 h	G	24 h	72 h	G
*CCL5* ^1,2,3^	C-C motif chemokine ligand 5	4.768	0.055	↓	0.828	3.317	↑	1.072	2.224	↑
*IL-18* ^1,2,3^	Interleukin 18	1.757	3.505	↑	3.043	1.367	↓	2.863	2.496	↓
*ICOSLG* ^1,2,3^	Inducible T-cell co-stimulator ligand	0.000	2.846	↑	3.070	0.176	↓	1.052	0.919	↓
*MAP4K1* ^1^	Mitogen-activated protein kinase kinase kinase kinase 1	2.409	1.045	↓	0.000	2.258	↑	0.031	2.740	↑
*LTB* ^1^	Lymphotoxin beta	6.443	2.071	↓	2.140	4.072	↑	0.097	3.354	↑
*KLRD1* ^1^	Killer cell lectin-like receptor D1	2.177	1.035	↓	0.000	2.207	↑	0.019	0.000	↓
*CXCL6* ^2,3^	C-X-C motif chemokine ligand 6	1.371	0.034	↓	0.000	3.249	↑	0.038	3.392	↑
*C3AR1* ^2^	Complement component 3a receptor 1	1.454	0.011	↓	0.000	1.185	↑	0.012	1.533	↑
*SELL* ^2^	Selectin L	3.421	0.032	↓	0.000	2.585	↑	0.036	1.921	↑

Hallmark gene sets of all pulp capping materials presented allograft rejection, inflammatory response, and TNF-α via the NF-κB signaling pathway (Figure 3B). Hallmark gene sets: ^1^, allograft rejection; ^2^, inflammatory response; and ^3^, TNF-α via the NF-κB signaling pathway. G refers to the gradient between 24 h and 72 h. ↑, (+) gradient over time; ↓, (−) gradient over time.
